# Successful treatment with femoro-femoral venovenous extracorporeal membrane oxygenation in traumatic tracheal injury: a case report

**DOI:** 10.1186/s13019-022-01991-8

**Published:** 2022-09-21

**Authors:** Haein Ko, Sang Gi Oh, Sang Yun Song, Kyo Seon Lee, Do Wan Kim

**Affiliations:** Department of Thoracic and Cardiovascular Surgery, Chonnam National University Hospital, Chonnam National University Medical School, 42 Jebong-Ro, Dong-gu, Gwangju, 61469 Republic of Korea

**Keywords:** Extracorporeal membrane oxygenations, Trauma, Airway management

## Abstract

**Background:**

Traumatic tracheal injury is a rare type of trauma. In this type of injury, catastrophes may occur owing to a failure to secure the patient's airway. Extracorporeal membrane oxygenation (ECMO) is rescue therapy available for the treatment of urgent cardiorespiratory distress until the patient's vital signs have stabilized. The various applications of ECMO configurations have expanded the scope for this therapy.

**Case presentation:**

We describe the case of a 66-year-old man with tracheal rupture with thyroid cartilage fracture due to cultivator handle who was treated with veno-venous ECMO. This case reflects the role and limitations of veno-venous ECMO, in which patient survival was possible with a bi-femoral configuration while also ensuring a clear airway.

**Conclusion:**

We shared our experience with bi-femoral veno-venous ECMO as a therapeutic option to contribute to choosing an appropriate approach. Based on our review of the literature, the present case was an uncommon report of survival after tracheal rupture due to trauma without other complications.

**Supplementary Information:**

The online version contains supplementary material available at 10.1186/s13019-022-01991-8.

## Background

Airway injury is a rare but life-threatening damage that may occur after blunt and penetrating trauma to the neck and chest, as well as procedures such as endotracheal intubation and tracheostomy. While the estimated incidence of tracheobronchial injury (TBI) among trauma patients is 0.5–2%, the true incidence remains unknown as many trauma victims with TBI die at the accident scene [1]. The diagnosis of TBI starts with high suspicion in patients with dyspnea and respiratory distress. Airway management is always the first priority in all such patients. As acute desaturation and severe hypoxia occur, rapid sequence intubation is frequently performed. The treatment in most cases of TBI is definitive surgical repair of the injured airway; however, superficial TBI can be treated conservatively [2]. Bronchoscopy is recommended for evaluation of the injury site and severity to make a treatment plan. However, performing bronchoscopy is difficult in patients with unstable hemodynamics and inadequate ventilation status. Additionally, poor visualization of the airway owing to massive bloody secretions and debrids make it difficult to perform accurate examination. Thus, large numbers of patients are transferred to the operating room for surgical exploration and injury repair. As oxygenation support is essential until the airway repair is completed, veno-venous extracorporeal membrane oxygenation (VV-ECMO) may be a reasonable life-saving option and as a short-term bridge to definitive surgery. In this case, we applied VV-ECMO with femoro-femoral configuration in a patient who was unable to receive appropriate ventilation after traumatic tracheal transection.

## Case presentation

**Fig. 1:**
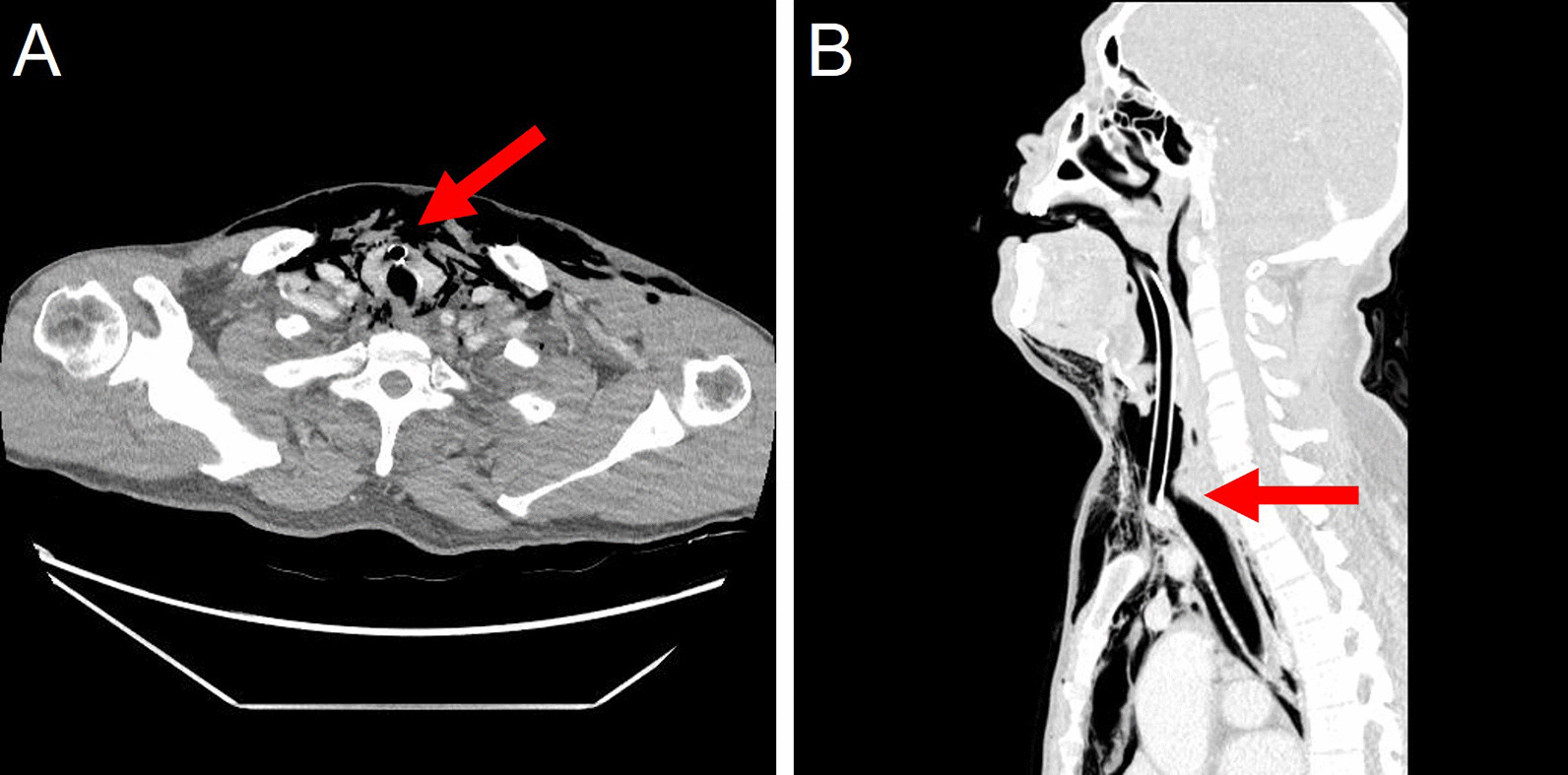
Computed tomography finding during the initial diagnostic period. **a** Intubation tube protruding toward the anterior side of the fractured trachea (red arrow); transverse view. **b** Intubation tube protruding toward the anterior side of the fractured trachea (red arrow); sagittal view

**Fig. 2:**
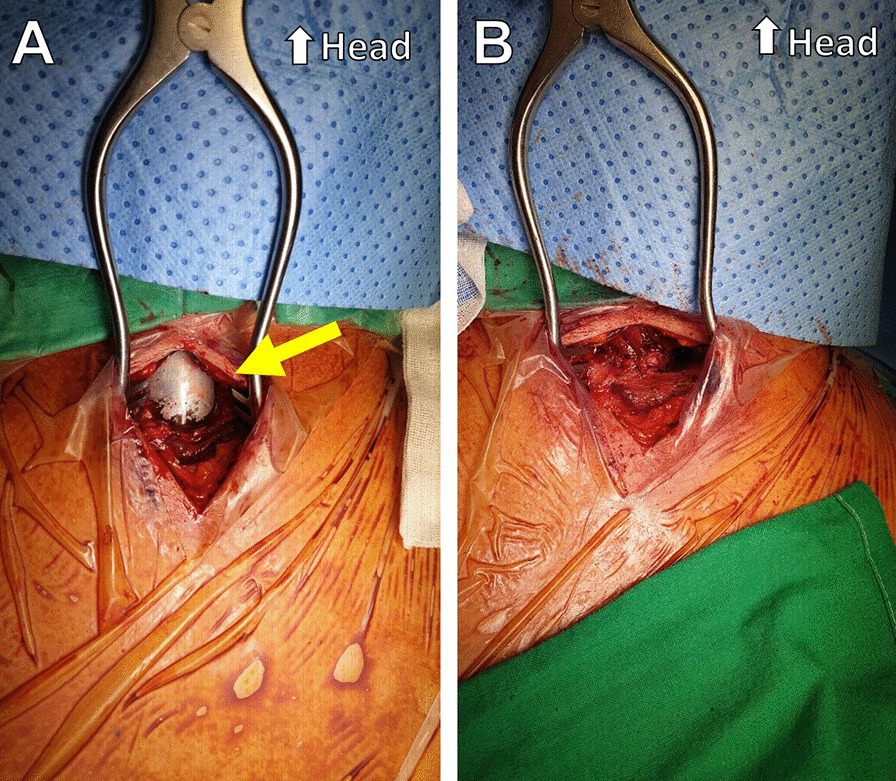
Intraoperative finding. **a** Preoperative view (yellow arrow). **b** Postoperative view after repair

**Fig. 3:**
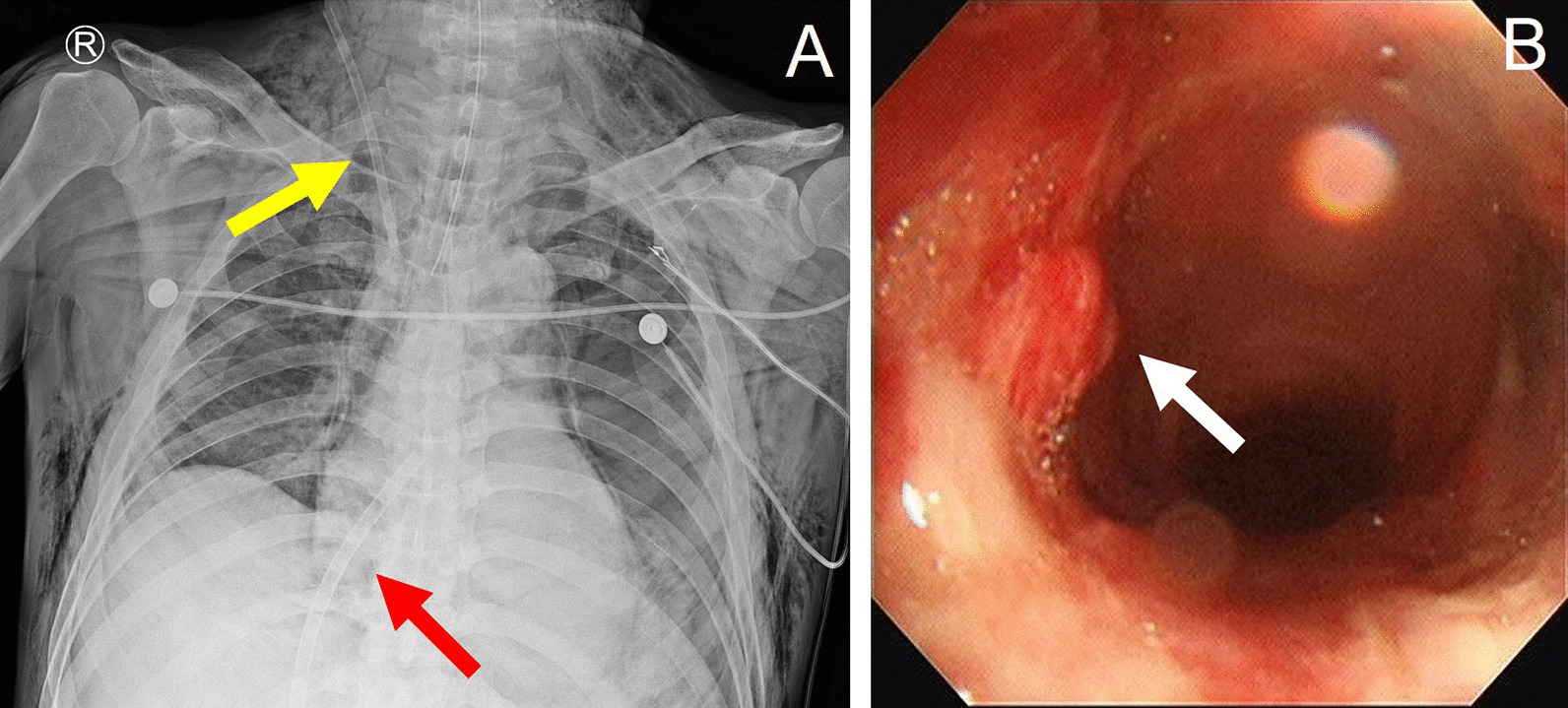
Postoperative finding. **a** Postoperative X-ray; yellow arrow: an advanced venous access catheter. (Arrow multi-lumen access catheter 9Fr, Teleflex, USA), ARROW® MAC (Multi-Lumen Access Catheter) red arrow: ECMO drain catheter. (MAQUET PLS, NJ, USA). **b** On the third day after surgery, bronchoscopy bronchoscopic finding. White arrow: injury site

**Fig. 4:**
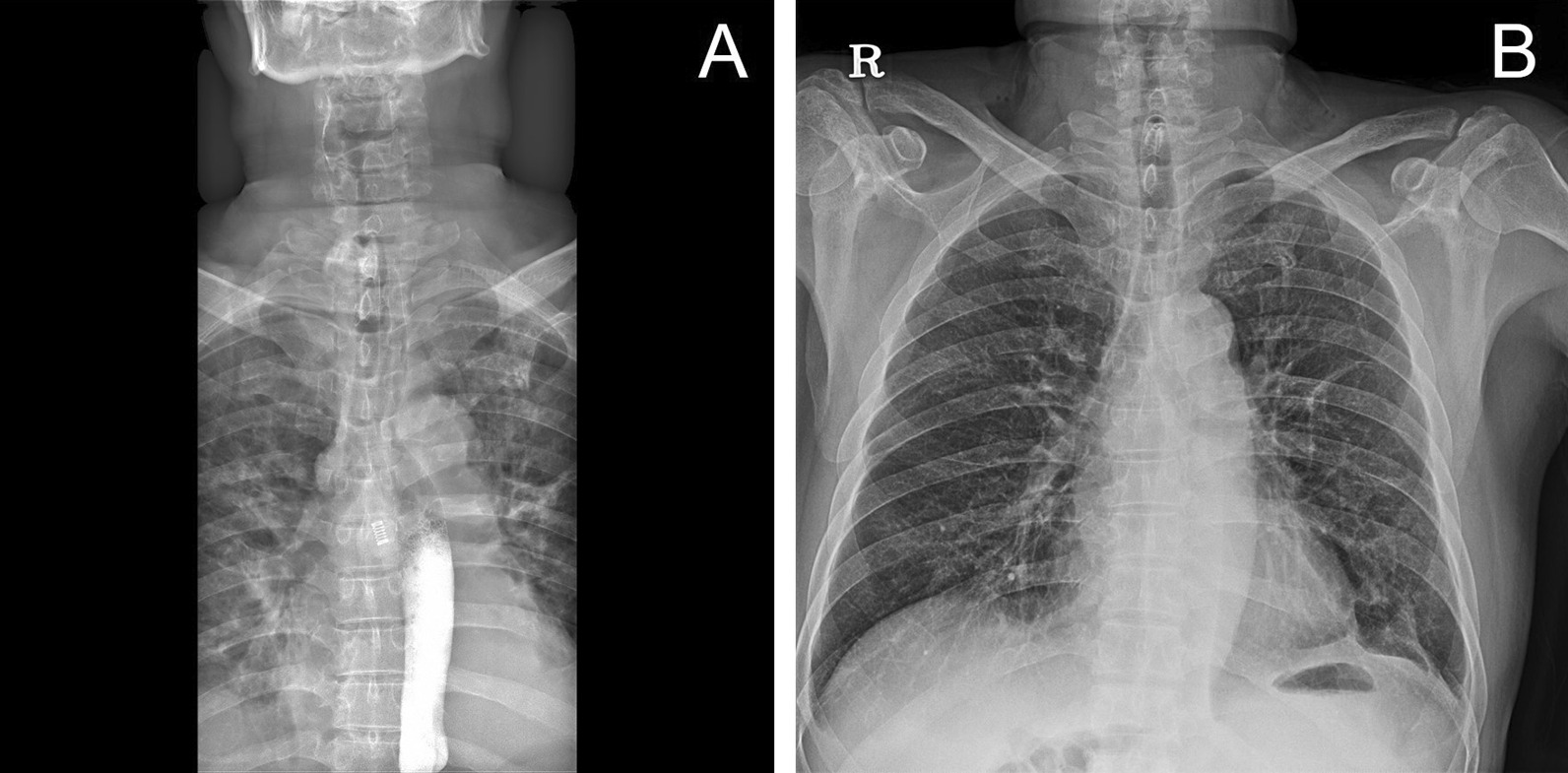
Findings at discharge. **a** Postoperative pharyngoesophagogram. **b** On the discharge day, Chest X-ray finding

A 66-year-old man with a history of arterial hypertension was referred to our hospital with an open wound on his anterior neck due to a cultivator accident. His arterial systolic blood pressure and heart rate were 130/80 mmHg and 80/min, respectively. His respiratory rate and SpO_2_ were 24/min and 85%, respectively, under a non-rebreathing mask with 15L/min oxygen. He was immediately intubated for dyspnea and hypoxemia. Chest and neck computed tomography (CT) to determine the degree of injury revealed thyroid cartilage fracture to the 3rd ring of the tracheal transection with extensive subcutaneous emphysema (Fig. 1). After bagging, the emphysema worsened, with the intubation tube protruding toward the anterior side of the fractured trachea. While preparing for emergency surgery, the patient’s SPO_2_ dropped to 70% and cyanosis was observed, with an O_2_ partial pressure of 40 mmHg. In addition, his mental status changed and he became drowsy. Although urgent tracheostomy or cricothyroidotomy were considered, it was not possible to perform them because the emphysema of his anterior chest was too extensive. Adequate ventilation was compromised owing to extrusion of the intubation tube; therefore, we decided to perform VV-ECMO. Given the risk of accessing the internal jugular vein because of the subcutaneous emphysema, we cannulated both femoral veins. The right femoral vein was used for venous drainage (23 French) while the left femoral vein was used for venous return (17 French) (MAQUET PLS, NJ, USA). After ECMO application, the patient’s saturation was maintained at > 90%. He was transferred to the operation room for surgery. After making a midline longitudinal incision on the anterior neck to expose the injured trachea, we confirmed tracheal transection and that the posterior membranous portion was intact (Fig. 2A). We withdrew the endotracheal tube (ETT) to the epiglottis level and adjusted the alignment of the trachea. We then advanced the ETT to the upper 1 cm of the carina and confirmed tidal volume recovery. Tracheal transection was repaired by end-to-end anastomosis with 4–0 Vicryl interrupted suture. The operation was completed after confirming that there was no air leakage of the surgical site (Fig. 2B). After being transferred to the intensive care unit, the patient’s respiratory function was well recovered and ECMO weaning was successful on the day following surgery (Fig. 3A). We did not start anticoagulation therapy owing to the risk of bleeding at the injury site; instead, the pump speed was maintained at high revolutions per minute (> 3500). On the third day after surgery, bronchoscopy was performed to confirm that the lesion was intact (Fig. 3B). The patient underwent percutaneous tracheostomy on the fourth tracheal ring using a bronchoscopic guide on the seventh day after surgery. After being weaned from the ventilator, the patient was transferred to the ward on the ninth day after surgery. We also confirmed that he had no accompanying injury or swallowing difficulty by pharyngoesophagram (Fig. 4A). The patient was discharged with comfortable respiration without additional oxygenation support on day 29 (Fig. 4B) and had received outpatient follow-up without severe complications for 12 months.

## Discussion

Traumatic airway injury is a rare type of trauma that is potentially life-threatening and occurring in less than 4.5% of cases [3]. It is usually seen in multiple-trauma patients and may be unrecognized and undertreated owing to its rarity [1]. Dyspnea and respiratory distress occur in most patients with TBI, with subcutaneous emphysema, pneumomediastinum, and pneumothorax also common findings in TBI. If possible, spontaneous breathing should be permitted as positive ventilation can exacerbate air leaks and rapidly worsen symptoms caused by airway injury [4]. Blind intubation may dislocate fractured cartilage or entirely disrupt a partial tracheal transection, leading to complete airway obstruction. The ideal initial management of TBI is pre-oxygenation followed by awake flexible bronchoscopy for the evaluation of airway injuries and safe endotracheal intubation [5]. However, in the clinical setting, rapid sequence intubation is frequently performed as there is difficulty in performing bronchoscopy based on a patient’s urgent clinical condition and resource limitations. CT is a good diagnostic tool in that it provides information on trachea transection, tracheal ring fracture, and associated injuries. The treatment of TBI should be individualized based on clinical manifestations and the injury extent and severity. While many cases of TBI eventually require definitive surgery, some minor injuries can be treated conservatively with follow-up bronchoscopy [6].

Tracheostomy or cricothyroidotomy directly through the fractured tracheal ring is another option for securing the airway in cases in which initial airway management fails. However, these procedures were not available in the present case as the subcutaneous emphysema was too extensive to approach the transected trachea with good visualization. As the patient’s hemodynamics began to worsen, we applied VV-ECMO considering the low probability of his survival without providing immediate oxygenation. As VV-ECMO support started, his condition stabilized enough to proceed with definitive surgery. If the decision had been delayed, there would have been no chance to stabilize the patient.

ECMO is currently considered a good and safe option for complex surgical cases and in patients with near-total airway occlusion [7]. The uses of ECMO in airway surgery range from reconstruction of tracheal stenosis to the repair of iatrogenic tracheal rupture. One case report described the successful repair of tracheal transection with VV-ECMO support [8]. In VV-ECMO, the jugular-femoral configuration is the treatment of choice for its low recirculation rate, which provides more efficient oxygenation and carbon dioxide clearance [9].However, in an emergency, aseptic painting must be performed in two separate locations, and ultrasound is often required; thus, it is sometimes not perfect in terms of expediting treatment [10]. In the present case, bi-femoral VV-ECMO was instituted as an alternative technique because the subcutaneous emphysema was too extensive for rapid cannula insertion. As subcutaneous emphysema accompanies in up to 87% of TBI cases [1], the femoro-femoral configuration should be considered when developing a cannulation strategy. The femoro-femoral configuration may be sufficient for temporary support as a short-term bridge to definitive surgery. Likewise, this configuration may be a reasonable option when accessing the jugular vein is difficult for other reasons such as severe edema, severe obesity, or neck site infection.

## Conclusion

In conclusion, we describe the acceptable clinical outcomes of a longitudinal injury from the thyroid cartilage to the 3rd tracheal ring that was treated with femoro-femoral VV ECMO, which is known to be ineffective. We completed the repair of high-level airway injury without severe complications or requirement for additional interventions. This case confirmed the effectiveness of ECMO in airway injury and expanded the indication for VV-ECMO to airway trauma in which conventional airway management cannot provide adequate ventilation. Based on our review of the literature, this is a rare case report of successful repair of tracheal injury with femoro-femoral VV-ECMO in airway trauma.

## Supplementary Information

**Additional file 1:** Chest CT.

**Additional file 2:** Postoperative pharyngoesophagogram.

## Data Availability

As this paper is a case report, all generated or analyzed data are included in this article.

## Abbreviations

TBI: Tracheobronchial injury
VV-ECMO: Veno-venous extracorporeal membrane oxygenation
CT: Computed tomography
ETT: Endotracheal tube
